# Spike-Interval Triggered Averaging Reveals a Quasi-Periodic Spiking Alternative for Stochastic Resonance in Catfish Electroreceptors

**DOI:** 10.1371/journal.pone.0032786

**Published:** 2012-03-05

**Authors:** Martin J. M. Lankheet, P. Christiaan Klink, Bart G. Borghuis, André J. Noest

**Affiliations:** 1 Experimental Zoology, Wageningen University, Wageningen, The Netherlands; 2 Helmholtz Institute, Utrecht University, Utrecht, The Netherlands; 3 Netherlands Institute for Neuroscience, Royal Netherlands Academy of Arts and Sciences, Amsterdam, The Netherlands; 4 Janelia Farm Research Campus, Howard Hughes Medical Institute (HHMI), Ashburn, Virginia, United States of America; Dalhousie University, Canada

## Abstract

Catfish detect and identify invisible prey by sensing their ultra-weak electric fields with electroreceptors. Any neuron that deals with small-amplitude input has to overcome sensitivity limitations arising from inherent threshold non-linearities in spike-generation mechanisms. Many sensory cells solve this issue with stochastic resonance, in which a moderate amount of intrinsic noise causes irregular spontaneous spiking activity with a probability that is modulated by the input signal. Here we show that catfish electroreceptors have adopted a fundamentally different strategy. Using a reverse correlation technique in which we take spike interval durations into account, we show that the electroreceptors generate a supra-threshold bias current that results in quasi-periodically produced spikes. In this regime stimuli modulate the interval between successive spikes rather than the instantaneous probability for a spike. This alternative for stochastic resonance combines threshold-free sensitivity for weak stimuli with similar sensitivity for excitations and inhibitions based on single interspike intervals.

## Introduction

The generation of neural action potentials involves a fundamental threshold nonlinearity that often interferes with processing small-amplitude stimuli. Although in some cases thresholds could help to suppress unwanted noise, they often limit sensitivity in sensory systems by blocking sub-threshold modulations of activity. Comparable problems are observed in many fields of science and technology and great progress has been made in understanding how systems with an inherent threshold can be optimized to provide optimal differential sensitivity. The solution adopted by a wide range of systems consists of exploiting stochastic resonance, i.e., the addition of an optimized amount of noise that induces a moderate, highly irregular, spontaneous background activity. Stimulus-evoked modulations of this spontaneous activity then provide threshold-free detection [1], [2], [3]. Stochastic resonance theory explains that noise is essential for linearization and actually helps rather than hinders detection [4]. Most neurons in the central nervous system operate as predicted by this theory [4], [5]. Here we show that electroreceptors of the passively electric Brown Bullhead catfish (*Ictalurus nebulosus*) have adopted a radically different strategy: The spike generation mechanism is set to produce high-rate quasi-regular spiking which not only prevents the underlying nonlinearity from hindering small-signal processing but also implies that stimuli do not modulate the probability of spike occurrence, but primarily the duration of interspike intervals.

Catfish electroreceptors consist of 10–20 sensory cells in the lumen of an ampul, converging onto one or two afferents with excitatory synapses [6]. Catfish live in murky waters and use their electroreceptors to detect electric fields generated by potential prey. These electric fields are extremely weak [7] and steeply decline in strength with increasing distance to the source. Electroreceptor performance therefore directly limits the distance over which prey can be detected, suggesting that they should be optimally adapted to sensing, encoding and processing signals relevant to this task. Whereas e.g. the electroreception ampullae of Lorenzini in sharks operate in accordance with stochastic resonance theory [8], the ampullary electroreceptors of *Ictalurus nebulosus* employ a different type of behavior. They exhibit a high spontaneous activity (about 50 spikes/s) that is far more regular than the typical random Poisson process one would expect for noise-driven spontaneous activity [9]. Furthermore, they show no signs of the sub-threshold modulations encountered in ampullae of Lorenzini [10].

The presence of highly regular spontaneous activity contradicts stochastic resonance as a solution to overcome threshold nonlinearities. At first sight, this might suggests that the spike-generation nonlinearities could hamper linear processing of realistically small signals, but this is contradicted by earlier experiments that characterized the electroreceptor's near linear filter properties [11]. This raises the question of how electroreceptors achieve their highly effective detection of prey by means of weak electrical stimuli [12].

To solve this problem we tested the hypothesis that the electroreceptor's spontaneous activity is not noise-driven, but caused by a DC bias current [13] that repetitively and deterministically drives the afferent neuron to threshold. We introduce an analysis technique that allows us to distinguish such quasi-periodic spiking from the typical behavior expected from stochastic resonance theory. Our analysis is a crucially modified version of a common reverse correlation technique that uses spike triggered averaging to estimate filter properties. Spike triggered averaging (STA) [14], [15] recovers the average stimulus profile that precedes a spike. Using Gaussian noise stimuli, STA reveals the system's transfer properties and it provides a good estimate of sensory filter properties preceding spike generation for neurons that operate in a stochastic resonance regime [16]. In such a regime, each spike conveys a similar message and the presence and timing of individual spikes encodes relevant information.

In the quasi-periodic spiking regime, on the other hand, spike presence is determined by the spike generator itself rather than by the input signals. Under these conditions, input signals act by modulating the duration of interspike intervals. To characterize signaling in this regime we calculate Spike Interval Triggered Averages (SITAs), a reverse correlation technique that triggers on pairs of spikes separated by specific intervals, rather than on single spikes. For neurons operating in the stochastic resonance regime, SITA curves for different interspike interval durations match the classic spike triggered average. For neurons operating in the quasi-periodic firing regime, we show that the SITA curves depend heavily on spike interval. For very long and short interspike intervals, the SITA curves become almost sign-inverted copies of each other, suggesting that the message that a spike conveys varies with the duration of the preceding interspike interval.

Numerous studies, starting with de de Ruyter van Steveninck and Bialek [17], have shown that different spike patterns may be correlated with different stimulus features (see also [18], [19], [20]). Oswald et al. [19], [20] for example revealed different feature triggered averages in neurons operating in a spike-bursting regime. Neiman and Russell [21], [22] studied the effect of stochastic oscillations on coding in paddlefish electroreceptors, and there is ample evidence for functional consequences of nonrenewal spike train statistics on neural coding [23], [24]. For catfish electroreceptors, using spike-interval triggered averages we find a pattern of results that clearly differs from these and other effects described previously. SITA analysis reveals major differences in spike generation from that of a Poisson spike generator or of a neuron operating in stochastic resonance mode. Using a simple leaky integrate and fire (LIF) model [25], [26], [27], [28], [29] in combination with linear filters, we show that this electroreceptor behavior can be explained by an interaction between a linear pre-filter and a dynamic spike generator operating in the quasi-periodic regime. This straightforward model reproduces the measured SITA curves accurately, while at the same time reproducing the near perfect linear behavior for sinusoidal stimuli [11].

## Results

**Figure 1:**
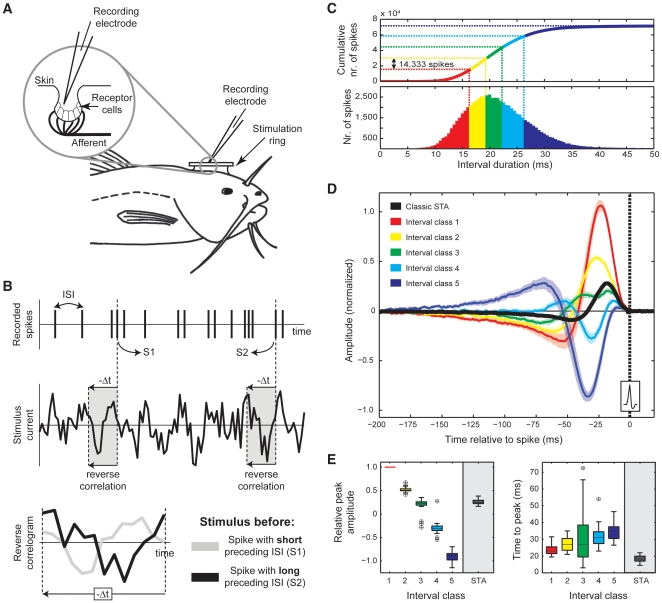
Spike interval triggered averaging (SITA). (A) The recording setup. Stimulation currents are applied locally through a stimulation ring, while spikes from afferent are recorded from within the lumen of the electroreceptor. (B) Example of the reverse correlation technique. For each recorded spike the stimulus shape is analyzed in a directly preceding time interval (-Δt). For each measurement about 75,000 spikes were grouped in 5 classes with equal numbers of spikes in each class, based on the cumulative distribution of interspike interval durations (intervals preceding spikes). (C) Interspike interval distribution (bottom panel) and cumulative interspike interval distribution (top panel). Spike-triggered averages were generated for each class separately. (D) Overview of spike interval triggered averages (SITAs) for 26 electroreceptors recorded in 20 catfish. Colors indicate the different interval classes as shown in (C). Confidence intervals for each class represent ±2*SEM. The overall STA is shown in black and the moment of the trigger spike is represented by the dashed line at T = 0 ms. (E) Left hand panel: Distributions of peak amplitude values, normalized to the amplitude for the shortest interval class. Right hand panel: distribution of peak latencies.

Figure 1 illustrates how SITAs are constructed in a reverse correlation experiment. We recorded spikes from electroreceptors in response to Gaussian white noise stimulation (Fig. 1A) and constructed STAs of the stimulus-shape preceding spikes (Fig. 1B) as a function of interval duration. To this end, recorded spikes were divided in five classes based on the duration of their preceding interspike interval (Fig. 1C). Since the total number of spikes was about 75,000 and we divided spikes in 5 equally sized classes, each class consisted of approximately 15,000 spikes. SITA analysis is a generalization of the standard STA (which is the average of all SITA curves), and reveals to what extent stimulus patterns correlate with interval durations, rather than spike timings. Differences between SITA curves for different spike-interval durations imply additional structure in the spike-triggered ensemble that is not picked up by conventional STA analysis.

**Figure 2:**
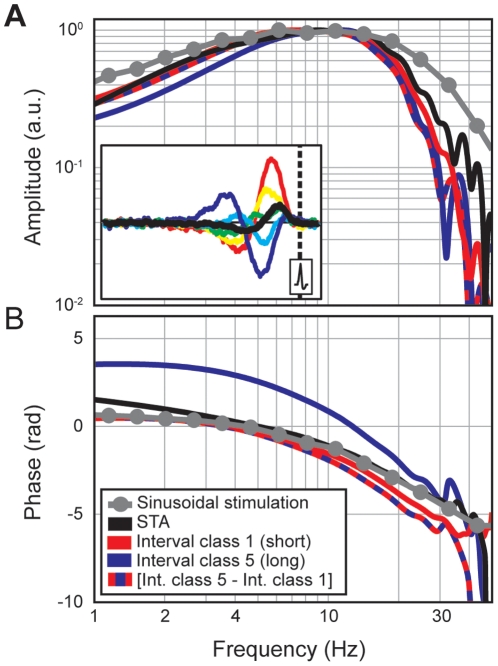
Bode plots comparing reverse correlation data and sinusoidal stimulation data for an example electroreceptor. (A) Amplitudes, (B) Phases. Grey lines with symbols show experimental measurements for sinusoidal stimulation. The other lines are based on Fourier transforms of STA and SITA data (see legend). The inset in (A) shows the example SITAs from the reverse correlation data that were used for these Fourier transforms.

Figure 1D shows group results of SITA analysis for 26 electroreceptors recorded in 20 catfish. It is immediately clear that curves for different interval durations are very different. In fact, curves for short and long spike-intervals are roughly of opposite polarity. Excitations due to positive preceding stimuli correspond to short intervals whereas inhibitions due to negative stimuli correspond to long intervals. The fact that these effects are of opposite sign means that they largely cancel out in the classic, overall, STA (black curve). Confidence intervals and distributions of peak latencies and peak amplitudes (Fig. 1E) confirm that the results are highly consistent across recordings. Since the shape and frequency content of the different SITA curves is highly similar (Fig. 2), the observed differences between SITA curves cannot be related to different stimulus frequencies. Instead, they reflect a general effect across all frequencies: Model simulations will show that the observed effects can be explained by a single linear filter in combination with a spike generator operating in a quasi-periodic regime. Figure 2 also shows the nonlinear behaviour of catfish electroreceptors. For a linear system the STA reflects the system's impulse response, and its Fourier transform should reflect the linear transfer properties as measured with sinusoids. The example in Figure 2 shows that for catfish electroreceptors these two measures may yield rather different estimates of the filter properties.

The overall STA amplitude is, on average, a factor of 0.26 (±0.05 SD) smaller than the SITA amplitude for the shortest intervals. Because SITAs for long and short intervals are not only polarity inverted but also slightly shifted in time (Fig. 1E, time to peak plot) the shape and timing of the overall STA may differ substantially from that of its separate SITA components. Peak latency for the overall STA is, for example, on average 5.8 ms shorter than that for the shortest interspike intervals (Fig. 1E in red) and about 20 ms shorter than that for the longest interspike interval (Fig. 1E in blue). Amplitude, timing and shape of the STA are therefore to a large extent determined by opposite SITA shapes for long and short interspike intervals.

Differences between the overall STA and the SITA curves are even more evident if we compare the additional power (sum of squared signals) in the SITA curves relative to the power in the overall STA. For a SITA based on five classes the total mean variance across the five classes, in a time window of 100 ms preceding spikes, is about a factor of 10 larger than the total power in 5 random subdivisions of the STA. For subdivisions into a larger number of classes the difference grows asymptotically to a slightly higher value (about a factor of 12 for 16 classes) because it allows for more accurate estimates of the variation with interval duration. The large additional power in SITAs relative to the STA clearly supports the notion that the overall STA cancels out most of the interval-related variance in the spike-triggered ensemble. A description in terms of a single impulse response that matches the overall STA therefore misses contributions from different SITA components.

**Figure 3:**
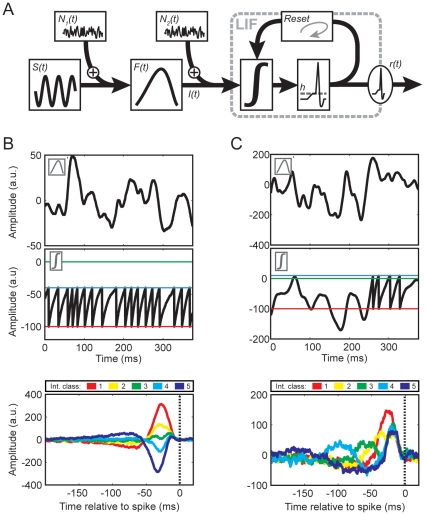
The Filter-LIF model. (A) Schematic diagram. The model consists of a linear band-pass filter (*F(t)*), taking the stimulus (*S(t)*) plus added noise (*N_1_(t)*) as its input, followed by a standard LIF spike generation mechanism. The LIF spike generator performs a leaky integration of the filter output plus a second noise source (*N_2_(t)*). This second noise source corresponds to a high frequency noise on the spike threshold. If the integrated signal crosses the threshold level (*h*), a spike is generated and the integrator is reset to a value of −100. (B) Model behavior for quasi-periodic spiking. (C) The behavior in a regime where spike generation is strongly driven by noise and external input signals. The linear filter stages were the same for both simulations, except for a gain factor. The top panels show examples of output signals from the linear filters. The second row displays the course of the ‘membrane potential’, including reset and post-spike recovery. The two regimes differ in the setting for the threshold (blue line) relative to the resting level (green line) and reset level (red line). For deterministic firing (B) the threshold is set well below the resting level, whereas for input-driven firing (C) it is set above the resting level. The bottom row panels represent the SITAs, with interval classes corresponding to the colors in Fig. 1.

To illustrate how SITA analysis distinguishes spike generation within the usual stochastic resonance regime from that within the quasi-periodic firing regime, we adopted a simple Leaky-Integrate-and-Fire (LIF) spike generation model [25], [26], [27], [28], [29] in combination with a linear pre-filter (Fig. 3A, see Methods for details). The LIF spike generator is a simplification of the Hodgkin-Huxley model [30] and incorporates only the mere basics of spike generation dynamics. In the absence of external stimulation the membrane potential exponentially recovers to a resting level. Whenever the membrane potential crosses the spiking-threshold an action potential is generated and the membrane potential is reset to a fixed, low level. The model operates in stimulus-driven (stochastic resonance) mode for resting levels below spiking-threshold (Fig. 3C) and in quasi-periodic firing mode for resting levels above the spiking-threshold (Fig. 3B). In the quasi-periodic setting the model produces results very similar to the electroreceptor measurements. In this regime, recovery of the membrane potential after a reset is sufficient for repetitive firing, which in turn causes the same clear reversals of SITAs that were also evident in the experimentally obtained SITAs. Modulatory effects of stimuli on quasi-periodic spike generation can thus explain SITA reversals: positive stimuli accelerate spike generation whereas negative stimuli (temporarily) postpone spike generation. SITAs of opposite polarity for long and short intervals therefore reflect one and the same linear pre-filter.

In the stimulus driven regime (Fig. 3C) the SITA-curve polarity inversions are absent, and individual traces largely resemble the classic STA. The higher noise levels in the simulated SITA curves of this regime are a consequence of the generally lower spike rate. We used the same linear filter properties, stimulus durations and stimulus dynamics for simulations with the two regimes, which naturally results in a larger number of spikes for quasi-periodic spiking than for purely stimulus-driven spiking.

While electroreceptors illustrate the surprising consequences of a quasi-periodic spike-generator, we also verified whether SITA analysis correctly picks up the more standard stochastic resonance type of spike-generation. To this end, we applied the technique to recordings from cat retinal ganglion cells, known to operate in the stimulus-driven regime [16], [31]. The random pixel arrays used in these studies were broadband in both the spatial and temporal domain to provide accurate estimates of linear response properties [16], [31]. The example curves in Figure S1 show great similarity to the model profiles in Figure 3C and lack the polarity inversions characteristic observed for the quasi-periodically spiking electroreceptors. Thus, in retinal ganglion cells each spike conveys the same type of information about the driving input, irrespective of spike-interval duration. In this case, the conventional STA provides a good estimate of the neuronal filter properties that precede spike generation [31], [32].

The LIF model reproduces both types of spiking behavior, depending on the threshold level relative to the resting level. Extensive model simulations in which we varied linear filter properties, thresholds, stimulus amplitudes and noise amplitudes revealed that the behavior in Figure 3B can only be obtained for deterministic, repetitively firing neurons with spiking-thresholds below the resting level. The LIF model in the stochastic resonance regime cannot reproduce this behavior. In the stochastic resonance regime, spikes occur when excitations drive the potential to threshold; inhibitions therefore remain invisible, unless followed by an excitation. On average, SITAs will thus show a short latency excitation, with a longer latency inhibition that is more pronounced for longer intervals (dark blue curve in 3C). Only the model in the quasi-periodic regime can reproduce the SITA results for electroreceptors. The interaction of linear pre-filtering with the dynamics of such a deterministic spike generator implies that stimuli primarily modulate the duration of interspike intervals, whereas the instantaneous probability for a spike is determined by spike generation dynamics rather than stimuli. Conventional STAs cancel out the variations with spike interval duration and are therefore blind to variations in the spike-triggered ensemble due to quasi-periodic spiking.

Our Spike Interval Triggered Average is fundamentally different from the Spike-Triggered Covariances (STCs) that are commonly used to recover multiple response components that might become superimposed or cancel out in the STA [14], [33]. Both STAs and STCs are based on the timing of single spikes and do not take interval durations explicitly into account. In contrast, the variation we describe depends critically on these spike-intervals. As a control, we also calculated STCs for our data, but we did not observe relevant eigenvectors beside the first eigenvector (the STA). This is in line with the main effect we observe; a sign reversal, which is irrelevant in an analysis of variance. While the Volterra kernel approach put forward by Marmarelis and co-workers [34], [35], [36] is especially sensitive to nonlinear summation of response contributions from different stimulus components, it makes no reference to the effects of spike history and interval duration either. Moreover these analyses provide a black-box type approach for filtering plus spike generation together without any reference to underlying mechanisms. Here we show that our SITA analysis actually reveals how spike generation interacts with linear pre-filtering to yield different types of behaviour.

**Figure 4:**
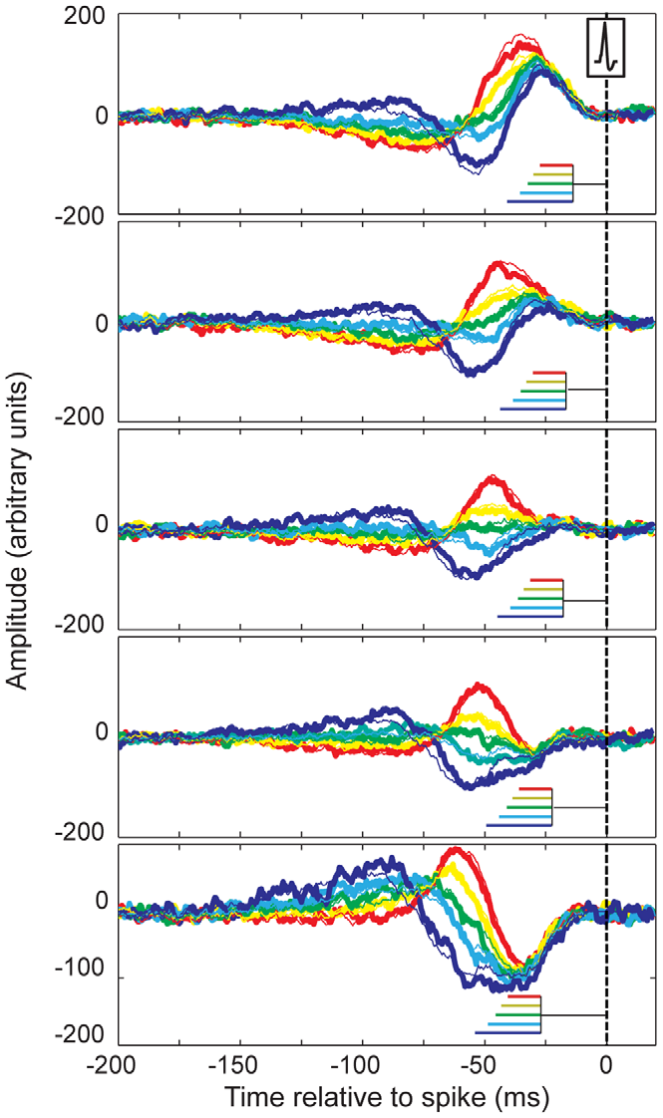
Two-interval SITAs. Each panel shows a single class of intervals subdivided according to the preceding interval. Interval durations are indicated by the insets in each graph. The black horizontal lines in the inset show the mean duration of the interval immediately preceding the trigger spike (at t = 0). The colored lines represent mean interval durations for the preceding intervals, with colors corresponding to the different curves. Color codes are similar to those in Figure 1. Thick lines correspond to measured data, thin lines to predictions based on linear summation of separate and independent SITAs for the two consecutive intervals. In calculating the linear sum of the SITAs for the first of the two intervals we used a time shift equal to the mean interval duration for the second interval (black horizontal line in insets). Linear predictions and actual measurements are highly similar, indicating that adding a second interval to the analysis provides no information that was not already present in the single interval analysis.

For fly H1-cells, which are sensory neurons involved in optic flow perception, it has been shown that additional information can be extracted from spike trains when more complex spike patterns are taken into account [17], [37]. The question therefore arises whether additional information involving multiple spike-intervals could still be hidden in the SITA curves for electroreceptors. To examine the possibility of SITAs reflecting an even more complex combination of multiple interval effects, we extended our analysis to two consecutive interspike intervals. We subdivided each interval class into another 5 subclasses according to the duration of the secondary, preceding spike-interval. With this subdivision, each separate curve is based on the combination of two spike intervals and the question is whether these two contributions simply add up linearly, independent of interval combination, or whether they show interactions. The results (Figure 4) demonstrate that the obtained patterns are nearly perfectly explained by linear combination of SITAs for consecutive intervals. The thick lines show SITAs for different combinations of interval durations, as indicated by the insets: The top panel shows data for a short interval preceded by different interval classes (color coded). Horizontal lines in the insets represent the mean interval durations. Thin lines in the graph show predictions for linear combinations of SITAs, constructed as the average of two SITAs, with time shifts equal to the mean duration of the interval preceding the trigger spike. The match might even further improve if we would use actual spike intervals rather then mean spike intervals for a class. The absence of interval-specific interactions demonstrates that extending the analysis to more than a single inter-spike interval yields no additional information. This is in line with the complete reset that follows the generation of a spike in our LIF model.

**Figure 5:**
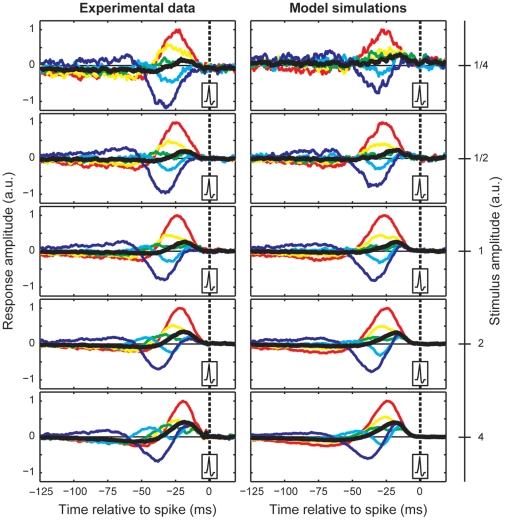
Reverse correlation results at different stimulus amplitudes. Experimental data (left column) and model simulations (right column). Model predictions were based on simulations with model parameters that were obtained by fitting the model to data from a standard reverse correlation experiment (third row of data, amplitude of 1). Noise amplitudes were varied by a factor of two between successive rows. Model simulations and actual measurements show very similar effects. At small stimulus amplitudes, SITAs for long and short intervals have similar shapes and comparable latencies. At higher stimulus amplitudes, shapes and latencies for different interval classes change drastically. Typically, inhibitory deflections become delayed relative to excitatory deflections and they may generate a short latency excitatory peak.

Based on the strong dynamic interactions between pre-filtering and spike generation one would expect clearly nonlinear behavior [38], [39]. This is indeed what we found for reverse correlation experiments. Different settings for stimulus amplitude greatly affected the shape of the reverse correlation functions, with curves becoming increasingly asymmetric for increasing stimulus amplitudes (Figure 5). For low amplitude stimuli the timing of different SITA components is quite similar, causing effective cancellation in the overall STA. For increasing stimulus amplitude the time to peak for the excitatory SITA (red curves in figure 5) becomes smaller whereas the peak latency of the inhibitory component consistently increases. Consequently, the relative amplitude of the overall STA, for instance, grows by a factor of more than two. Also, oscillations that are clearly present at high stimulus amplitudes are virtually absent at low stimulus amplitudes. At the highest stimulus amplitude the inhibitory curve for long interspike intervals (dark blue) shows a strong excitatory component at short latencies, which is absent for low stimulus amplitudes. Model simulations, in which parameters were fitted to a single stimulus amplitude (middle row) and held constant for other amplitudes, correctly predicted this type of nonlinearity and showed similar shifts in peak latency, oscillatory behavior and variation in relative STA amplitude.

**Figure 6:**
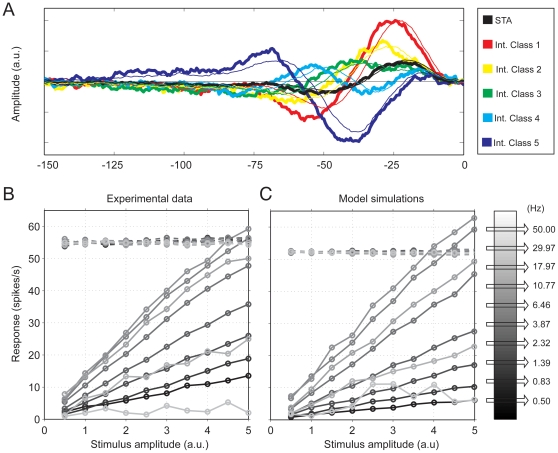
Example of model fit to SITA data and predictions for sine wave stimuli. (A) Comparison of experimental data (thick lines) and model fits (thin lines). (B) Experimental response amplitudes for sine wave stimuli of different amplitude (x-axis) and frequency (see legend). (C) Predictions for the same experiment based on the model fitted to the reverse correlation data in (A). Dashed lines in panels (B) and (C) represent mean spike rates, which are independent of stimulus frequency and amplitude; solid lines represent amplitudes of Post Stimulus Time Histograms (PSTH). Both experimental data and model predictions increase linearly with stimulus amplitude, as long as amplitudes stay below the mean spike rate. Higher amplitudes cause distortions due to clipping at zero spikes/s and compression at very high spike rates.

Quasi-periodic spiking combined with linear pre-filtering also consolidates inherent nonlinearities due to spike generation with nearly perfect linear behavior observed with sine wave stimuli [11]. The frequency transfer properties that we measured with sinusoids were highly similar to those reported by e.g. Bretschneider et al [11]. Low frequency slopes roughly corresponded to a half-order characteristic (mean slope 3.34±0.63 db/octave, for a frequency range of 0.5–3 Hz), low pass filtering was close to that of a third order filter (−14.78±6.4 db/octave, frequency range 20–40 Hz), and optimal frequencies were close to 10 Hz. Figure 6 shows that the model correctly reproduces the linearity for sine wave responses. Here, we fitted the model to SITA curves from a reverse correlation experiment (Figure 6A) and then simulated the responses to sine wave stimuli without adjusting any model parameters. The model (thin lines in Figure 6A) reproduces the recorded SITA curves quite well. Figure 6B shows recorded response amplitudes for sinusoidal stimuli that grow linearly with stimulus amplitudes for all temporal frequencies. The dashed horizontal lines show the corresponding mean spike rates, which are independent of stimulus amplitude and frequency. Model simulations (Fig. 6C) accurately reproduce this pattern of results. Indeed, within the quasi-regular spiking regime, the strong nonlinearity inherent in spike generation does not interfere with the nearly perfect linear behavior for sinusoids.

## Discussion

The combination of SITA analysis and model simulations demonstrates that electroreceptor afferents generate action potentials well outside the range of stochastic resonance. Their quasi-periodic spike-generating mechanism leads to a very different transformation of stimuli into spike trains. Specific temporal stimulus patterns (shape and polarity) correspond to different inter-spike interval durations, whereas the timing of spikes primarily depends on spike generation dynamics. For neurons operating in stochastic resonance mode, each single spike is informative about the degree to which a stimulus matched the filter-properties of the neuron [16], [18]. This (classic) assumption is only true for systems with Poisson type spike generation, for which separate SITA curves match the overall STA. The direct relation between inter-spike interval duration and stimulus statistics, as it is shown in the different SITA curves for catfish electroreceptors, generalizes this notion for quasi-periodic spike generation.

Our objective and approach in the current study is different from recent decoding studies that employ, for instance, the GLM framework with optimized post-spike feedback [31], [32]. These studies have been highly successful in reproducing and predicting response properties of retinal ganglion cells, including the details of spike patterns and complex neuronal network effects. Our objective was different and two-fold: 1) to develop a simple analysis technique that allows us to estimate the impact of spike generation on Spike-Triggered Ensemble (STE) data, and 2) to explain the observed complex response behavior with a simple, though physiologically realistic, model. In the case of catfish electroreceptors this provides a valuable new insight in their functional architecture. In catfish electroreceptors the correlation between spikes and preceding stimuli is to a large extent determined by the interspike interval rather than just by the timing of spikes. It reveals the quasi-periodic nature of spike generation, which does not conform to a simple rate transformation. Applying SITA analysis thus reveals a major nonlinearity and greatly helps in elucidating the mechanisms underlying electroreceptor response properties.

It is often thought that strong interactions between linear input filters and spike-generation dynamics hamper the extraction of useful information from spike trains. However, rather than presenting a nuisance that hinders the decoding of spike trains, a quasi-periodic spike generator might actually offer several important advantages to the animal. Firstly, in contrast to the stochastic resonance mode it requires no additional noise to allow for threshold-free detection. Since intrinsic properties drive the afferent to cross the threshold, noise levels can be minimized. Secondly, because no excitations are required to reach threshold, quasi-periodic spiking allows for a detection mechanism that is equally efficient for excitations and inhibitions. Finally, it does not require estimating spike occurrence probabilities. In contrast to a standard rate code [40] it provides information at the shortest possible time delay of a single interspike interval. As such, it provides a continuous and instantaneous estimate of how much the input signal resembles the shape and polarity of a specific temporal stimulus pattern. It remains an open question, however, to what extent and how such information is used in generating representations at higher processing levels.

In the vestibular system, Sadeghi et al. [29] have studied information transmission by regular and irregular afferents. Despite lower gains, regular afferents transmitted more information than irregular afferents. This may very well correspond to a different neural code, comparable to what we demonstrate for regularly firing electroreceptors. Information transmission in regular vestibular afferents was found to be highly sensitive to jittering the timing of spikes. At first thought, this may seem to contradict the importance of spike interval duration over mere spike timing, but jittering individual spikes of course also affects spike interval durations, especially in regularly firing units. An affect of jittering spike timings is therefore not incompatible with spike interval coding as suggested by SITA analysis.

The LIF model includes both increments and decrements of the current driving the afferent membrane potential to threshold. Since there are no indications of inhibitory synapses [41] in catfish electroreceptors, we must assume that the synapse is continuously active. Positive stimuli then increase and negative stimuli decrease the rate of neurotransmitter release. Model simulations, however, show that these modulations are relatively small compared to the currents that are responsible for recovery of the afferent membrane potential after a reset. Tonic neurotransmitter release is therefore unlikely the main driving force for spontaneous activity. Instead, spontaneous activity mainly results from intrinsic dynamics of the spike generator. This would explain why sensitivity and spontaneous activity are not directly related [12]. Our model suggests that intrinsic properties of the spike generator account for spontaneous activity while synaptic activity modulates the speed of recovery after a spike.

Our model analysis further reveals that for regularly firing neurons the spike triggered average may not provide an accurate estimate of receptor filter properties. SITA curves and their resulting overall STA are significantly affected by spike generation dynamics. Because the STA is the average of SITA components that are polarity inverted and slightly shifted in time, STA shape and latency do not reflect filter properties in a simple, straightforward manner. Our analysis also reveals that neuronal filter properties cannot be recovered by selecting only the long interspike intervals [42]. In the quasi-periodic spike generation regime this would result in extreme errors. Also in the noise driven regime (see Figure S1) the selection of a subset of interspike intervals may drastically alter estimates of filter properties. The extensive SITA oscillations for long interspike intervals in particular are not related to any filter properties (see model simulations). Separating STAs into contributions from long and short spike intervals reveals how, and to what extent, spike generation mechanisms affect the spike-triggered response ensemble. SITA analysis thus provides the additional information required to separate filter kernels from spike generation dynamics.

For catfish electroreceptors the spike history effects are huge. Similar effects probably play a significant role in spike-history dependent variations of average current trajectories preceding spikes in rat motoneurons [43]. Large effects, albeit of a very different nature may also be observed for neurons operating in, for example, a spike-bursting regime [19], [20]. For other systems, as illustrated here with cat retinal ganglion cells the implications of spike generation may be less surprising. Data for cat retinal ganglion cells (Figure S1) do not show the pronounced reversals that we saw for electroreceptors, indicating that for these cells and conditions, spike generation is primarily stimulus(/noise)-driven. We simulated this type of behavior with a large input gain, and a threshold above the equilibrium potential. It should be noted though that for high input gains the exact threshold level becomes quite irrelevant because, relatively speaking, stimulus-induced fluctuations are much larger. In this regime the Filter-LIF model's behavior does not substantially differ from a Linear-Nonlinear Poisson (LNP) model. This is in line with the high predictive value of the latter type of model, or the GLM framework that includes history dependence [31], [32] and network effects for e.g. ganglion cells [31]. SITA analysis may however still be very useful for identifying effects of spike generation. In combination with model simulations it provides insight into the mechanisms that cause variations in the STE. The SITA analysis therefore provides additional information that cannot easily be recovered by e.g. STC analysis [14], [33], [44] or nonlinear Volterra or Wiener kernel analyses [34], [35], [36], [45], [46], [47].

The SITA technique may elucidate operating modes in a wide range of nonlinear dynamical systems. It provides a simple analysis method to distinguish quasi-periodic transitions from stimulus- and noise-driven transitions. Moreover, it reveals when and how recent events play an important role in the generation of a future event, a question central to many systems ranging from low level sensors to high level mechanisms underlying e.g. binocular rivalry [48], [49].

## Materials and Methods

### Recordings

We recorded from ampullary electroreceptors in the skin of the Brown Bullhead Catfish (*Ictalurus nebulosus*), a passively electric fish that uses electroreceptors to sense electrical fields such as those generated by potential prey [7]. The electroreceptors consist of a group of sensory cells (10–20) in the lumen of an ampul, which make excitatory synapses onto one or two afferents [6]. Spikes from single afferents were recorded by placing the tip of a tungsten microelectrode near the opening of an ampul. The electroreceptors have a maintained discharge rate of approximately 50 spikes/s and respond nearly linearly, and with band-pass characteristics, to small-amplitude sinusoidal stimuli [11].

Anesthesia was induced by 4 mg/l Ethomidate, dissolved in water, and maintained by half of this concentration in the experimental setup. Ethomidate blocks central processing and conveniently immobilizes the animal, without blocking responses of the peripheral nervous system. Animals were artificially respirated with a water flow of about 100 ml/min. Experiments lasted up to 6 hours, after which the animals were transferred to a recovery chamber where artificial respiration was maintained during recovery. All experimental procedures and animal handling were in line with University regulations and approved by the University's animal experiment review committee (Approval ID 2008.I.06.043, Dierexperimentencommissie Academisch Biomedisch Centrum, Utrecht University). Recordings were obtained in 20 catfish, weighing 200–750 g. During the recordings, the fish were held in a perspex tray in which rubber clamps gently pushed their head up onto a nose-rest through which aerated water was supplied. An adjustable overflow was used to control the water level and return excess water to the aquarium. Experiments were performed at a water temperature of 20 degrees Celsius. To avoid spike-sorting problems we only selected electroreceptors with single afferents for our recordings.

An Apple G4 computer with a National Instruments PCI 1200 data-acquisition board controlled the experiments. Custom-written software (in C) was used to simultaneously generate stimuli, record spikes and stimuli, provide online data analysis, and store all information for further offline analysis. Spike-times were obtained at a temporal resolution of 2,000 Hz.

### Stimuli and Stimulus protocol

Stimuli consisted of low frequency (0.1–100 Hz) fluctuations of electrical potential and were generated at a sample rate of 1,000 Hz. Computer-generated stimuli ranged in amplitude between −5 and 5 Volts at 12 bits resolution and were reduced to a suitable amplitude range at the site of the electroreceptor by means of attenuating resistor circuits adjustable from 0 to 80 dB in steps of 1 dB. A voltage-to-current converter was used to render stimulation currents independent of resistance. Stimulation currents passed through a 1 cm^2^ area of skin surface, located at the dorsal head region. They were applied by means of a circular stimulation electrode, placed about 1.5 mm above the skin and surrounded by an insulating rubber ring that prevented leakage of stimulation current directly to ground (surrounding medium). The rubber ring prevents any direct contact of the stimulation electrode with the skin, mimicking natural stimulation as good as possible. To assure perfect correspondence of stimulus and response timings, computer generated stimuli were re-recorded by feeding output signals back into the AD converter.

Once a stable recording of sufficient signal-to-noise ratio was obtained, we first adjusted the attenuator box to a level where full-range sinusoidal modulation produced a response amplitude roughly equal to the mean firing rate of the cell (about 50 spikes/s). Within this range response amplitudes vary linearly with stimulus amplitude. No attempt was made to calibrate the absolute strength of the stimulus for each electroreceptor and stimulus strengths are therefore reported in arbitrary units (a.u.). For each electroreceptor we measured frequency transfer properties with sine wave stimuli, reverse correlation functions with white noise, and, if time allowed, several additional experimental variations. Frequency transfer properties with sinusoidal stimulus modulations were measured in the range of 0.5 to 50 Hz, in 20 or 30 logarithmically spaced steps. Trials lasted 2 seconds and were separated by 0.2 s inter-stimulus periods without stimulation. The order of stimulus presentations was randomized within repetitions and we typically recorded stimuli for a total duration of at least 30 s. In some of the experiments, we extended the frequency range to 0.1–100 Hz and increased trial durations to 10 seconds. Comparison of data obtained for single receptors in different protocols showed no differences due to this increased frequency range. In a subset of the recordings, we also measured frequency transfer properties at a range of sinusoidal amplitudes.

In reverse correlation experiments the stimuli consisted of white noise, generated at 1,000 Hz and passed though a single first order low pass filter (in software) with a corner frequency of 50 Hz. Filtering increased the power in the appropriate frequency range and transformed white noise into Gaussian white noise. The 50 Hz high frequency limit for the noise stimulus roughly corresponds to the high frequency limit for electroreceptor responses. As a control, we measured reverse correlations at different cut-off frequencies for the low pass stimulus filter. At cut-off frequencies below 50 Hz, we observed significant changes in reverse correlation functions. Data for 50 Hz were however identical to those obtained for 100 Hz. White noise responses were recorded in trials of 30 seconds. We typically used 10 different seeds and repeated trials 5 times. This resulted in a total recording time of 25 minutes, yielding roughly 75,000 spikes.

### Data analysis

Mean spike rates, response amplitudes and phases for sinusoidal stimulation were obtained by fitting a sinusoidal function to the Post Stimulus Time Histogram (PSTH) at the frequency of stimulation. For phase calculations we also fitted a sinusoid to the recorded stimulus and subtracted the resulting phase shift (if any) from the response phase shift. For analyzing the spike-triggered ensemble, spikes were grouped in 5 classes containing equal numbers of spikes, based on the cumulative inter-spike interval distribution (Fig. 1c). Spike Triggered Averages (STAs) were then calculated for each interval class separately, thus creating Spike Interval Triggered Averages (SITAs). Obviously, the mean value of these individual SITAs is the conventional STA. We divided spikes in 5 equally sized classes, each consisting of about 15,000 spikes. Our choice for five classes is rather arbitrary. As a control we also calculated SITAs for larger numbers of classes. Increasing the number of classes results in a higher resolution for the variation with interval duration, but the main effects remain similar. Initial data-analysis, including the construction of PSTHs and SITAs, was done in C. Further analysis and comparisons of experimental and modeling data was done in MATLAB (The MathWorks, Natick, MA).

### Modeling dynamic interactions

To show how the observed SITAs may arise from dynamic interactions between linear filtering and a nonlinear dynamic spike generator we combined these effects in a simple, quantitative model (Fig. 2A). The model consists of a linear filter in combination with a spike generator of the leaky-integrate and fire (LIF) type, which is a common simplification of the Hodgkin-Huxley model for excitable membranes [26], [27], [28]. In contrast to a Poisson spike generator, a LIF model-neuron includes the essential dynamics of spike generation that we wish to incorporate. The dynamics of the membrane potential *V*(*t*) are expressed in Equations 1 and 2 and include a membrane time constant *τ* and a spike generation threshold *h*.
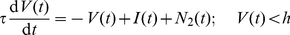
(1)

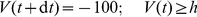
(2)


The *N_2_*(*t*) term describes a small uniform noise with a fixed RMS amplitude (±10). This noise source was introduced to obtain realistically smooth firing-rate functions. The main drive term *I*(*t*) is the filter output, defined formally as the convolution of the sensor's filter impulse response *F*(*t*) with mixture of the stimulus *S*(*t*), with gain *g_S_* and a front-end Gaussian noise term *N_1_*(*t*), with gain *g_N_* (Equation 3).
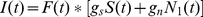
(3)


This noise term (*N_1_*) proved essential for reproducing proper interval distributions for both spontaneous and input-driven activity, as well as for scaling SITAs independent of the mean firing rate and specific SITA shape.

We modeled the system's band-pass filter properties with a series of first order high-pass and low-pass filters, representing both filtering in receptor cells and in the synapse onto the afferent axon. The high frequency fall-off is modeled with three first-order low-pass filters with the same corner frequency:
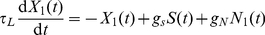
(4)

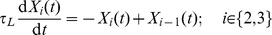
(5)The high-pass part, formally just a single ‘fractional-order’ stage [11], [50], can in our case be approximated by five in-parallel first-order stages:
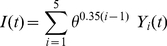
(6)

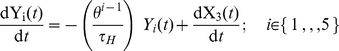
(7)


Finally, the model includes a time delay in the order of several milliseconds that allows for frequency independent delays. These include any delays between spike initiation and registration, e.g. due to the use of a window discriminator and/or threshold detection in the software.

Model simulations were run at 2,000 Hz, similar to the resolution of experimentally recorded responses. For numerical simulations, we used first order Euler integration with a time step of 0.5 ms, which proved sufficiently accurate. Model simulations were run using the same software, the same stimuli and the same procedures as for the physiological experiments. Model data were saved in the same format as the recorded data and analyzed using the same analysis routines.

We used the nonlinear fit procedure STEPIT [51] to fit the model to the data. The LIF spike generator had two free parameters: the time constant *τ* and the threshold level *h*. The formal parameters in *F*(*t*) were all tied to just 3 free parameters: the low-pass timescale, and two parameters that effectively determine the high-pass timescale and spectral slope (see Equations 6 and 7). The fit error was calculated as the sum of squared differences between experimental and model SITAs for 5 different interval classes, similar to the curves plotted in figure 1b. In addition, we added a small error term based on the difference in mean spike rates between model and experimental data. This assured that both the mean spike rate and SITAs were fitted correctly. Initial parameter values for the model fit were first estimated by trial-and-error. Correct optimization was checked by restarting the fit-procedure with different starting values. We did not analyze the reliability or confidence intervals for estimated parameters, because we were interested in the model's dynamic behavior rather than parameter quantification.

## Supporting Information

Figure S1 **SITAs for cat retinal ganglion cells.** Responses of ganglion cells were recorded in the optic tract of anesthetized and paralyzed cats. Stimuli consisted of binary, dark-light pixel arrays. The array measured 16×16 pixels and fully covered the cell's receptive field. Each pixel was modulated between light and dark levels in a unique random order. Stimuli were updated every second frame on a 100 Hz CRT display in front of the cat. Experimental and surgical procedures have been described in detail in previous publications [52], [53] and were in line with national and international guidelines. Reverse correlations were constructed for each pixel separately. Dark values were represented by a value of zero, light pixels by a value of 1 and correlograms describe the mean value at different stimulus-spike intervals, aligned with all spikes at time 0. The examples given correspond to a single pixel in the center of the receptive field of 8 Off center cells and 2 ON center cells. Apart from an inversion, due to the excitation by light off for an Off center cell, the pattern of results strongly resembles that seen in Fig. 3C. Notice that no attempt was made to fit model parameters in Fig. 3C to actual data. In Fig. 3C all parameter values were chosen equal to those in 3B, except for the spike threshold value and stimulus amplitude. It can be seen that SITAs for cat retinal ganglion cells show behavior similar to what is predicted by the stimulus-driven regime: a lack of inversions at short stimulus-spike intervals, combined with large oscillations for long inter-spike intervals. We refer to this regime as the stimulus-driven regime, because spike timings are largely determined by the filter output and to a lesser extent by the dynamics of spike generation. The present findings mainly concern the alternative, quasi-periodic regime, where spike generation is the dominant factor.
(TIF)
