# Correction: Spike-Interval Triggered Averaging Reveals a Quasi-Periodic Spiking Alternative for Stochastic Resonance in Catfish Electroreceptors

**DOI:** 10.1371/annotation/1997d0fd-63cf-4593-877b-604c3e7b524f

**Published:** 2012-06-12

**Authors:** Martin J. M. Lankheet, P. Christiaan Klink, Bart G. Borghuis, André J. Noest

The published affiliations of Bart G. Borghuis and André J. Noest are incorrect. The correct affiliations are:

Bart G. Borghuis 4 Janelia Farm Research Campus, Howard Hughes Medical Institute (HHMI), Ashburn, Virginia, United States of America.

André J. Noest 2 Helmholtz Institute, Utrecht University, Utrecht, The Netherlands. 

