# Supplementary figures and images for: Spike-Interval Triggered Averaging Reveals a Quasi-Periodic Spiking Alternative for Stochastic Resonance in Catfish Electroreceptors
Source: PLoS One. 2012 Mar 5;7(3):e32786. doi: 10.1371/journal.pone.0032786 (PMC3293861; doi:10.1371/journal.pone.0032786)

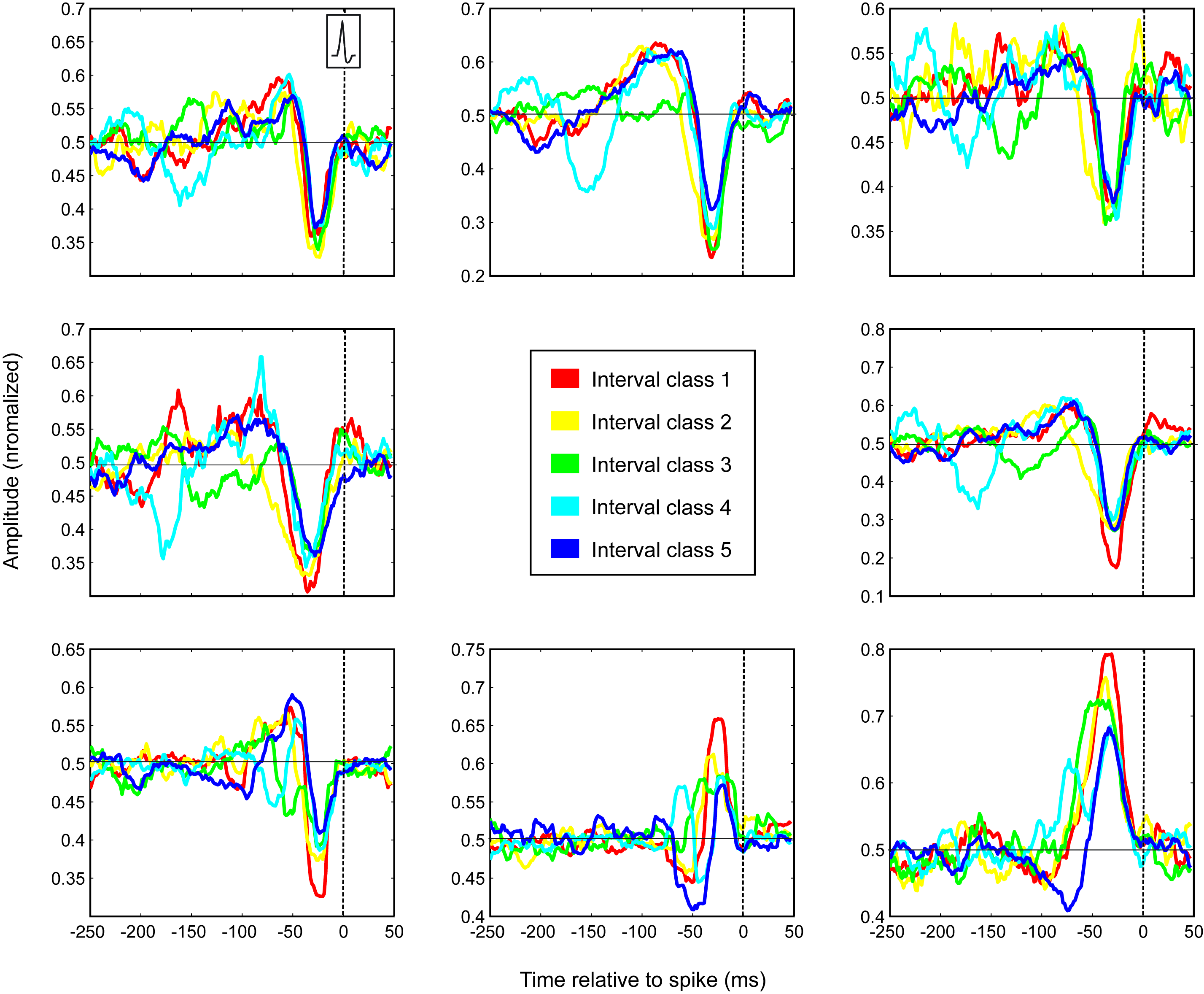

Supplement: Figure S1 — SITAs for cat retinal ganglion cells. Responses of ganglion cells were recorded in the optic tract of anesthetized and paralyzed cats. Stimuli consisted of binary, dark-light pixel arrays. The array measured 16×16 pixels and fully covered the cell's receptive field. Each pixel was modulated between light and dark levels in a unique random order. Stimuli were updated every second frame on a 100 Hz CRT display in front of the cat. Experimental and surgical procedures have been described in detail in previous publications [52], [53] and were in line with national and international guidelines. Reverse correlations were constructed for each pixel separately. Dark values were represented by a value of zero, light pixels by a value of 1 and correlograms describe the mean value at different stimulus-spike intervals, aligned with all spikes at time 0. The examples given correspond to a single pixel in the center of the receptive field of 8 Off center cells and 2 ON center cells. Apart from an inversion, due to the excitation by light off for an Off center cell, the pattern of results strongly resembles that seen in Fig. 3C. Notice that no attempt was made to fit model parameters in Fig. 3C to actual data. In Fig. 3C all parameter values were chosen equal to those in 3B, except for the spike threshold value and stimulus amplitude. It can be seen that SITAs for cat retinal ganglion cells show behavior similar to what is predicted by the stimulus-driven regime: a lack of inversions at short stimulus-spike intervals, combined with large oscillations for long inter-spike intervals. We refer to this regime as the stimulus-driven regime, because spike timings are largely determined by the filter output and to a lesser extent by the dynamics of spike generation. The present findings mainly concern the alternative, quasi-periodic regime, where spike generation is the dominant factor. (TIF) [file pone.0032786.s001.tif]
